# Impact of the Glycemic Level on the Salivary Proteome of Middle-Aged and Elderly People With Type 2 Diabetes Mellitus: An Observational Study

**DOI:** 10.3389/fmolb.2021.790091

**Published:** 2021-12-10

**Authors:** Shu Yuan Jia, Yan Ling Zhang, Xiang Yu Sun, Chao Yuan, Shu Guo Zheng

**Affiliations:** ^1^ Department of Preventive Dentistry, Peking University School and Hospital of Stomatology, National Center of Stomatology, National Clinical Research Center for Oral Diseases, National Engineering Laboratory for Digital and Material Technology of Stomatology, Beijing, China; ^2^ Department of Periodontology, Peking University School and Hospital of Stomatology, National Center of Stomatology, National Clinical Research Center for Oral Diseases, National Engineering Laboratory for Digital and Material Technology of Stomatology, Beijing, China

**Keywords:** Data-independent acquisition, mass spectrometry, type 2 diabetes mellitus, salivary proteome, salivary secretion, oxidative stress

## Abstract

Type 2 diabetes mellitus (T2DM) is an increasing global public health concern, but its impact on the salivary proteome is still unclear. To evaluate the effect of glycemic levels in middle-aged and elderly individuals with T2DM on salivary proteomics, we compared the differences by liquid chromatography tandem mass spectrometry (LC–MS/MS). Unstimulated whole saliva samples from 8 T2DM patients with good glycemic control (G group, HbA1c <6.5%) and 16 patients with poor control (P group, HbA1c ≥6.5%) were analyzed by LC–MS/MS in the data-independent acquisition mode (Clinical register number: ChiCTR1900023582.). After functional annotation, cluster analysis and receiver operating characteristic (ROC) curve analysis were carried out to screen and evaluate candidate proteins. A total of 5,721 proteins were quantified, while 40 proteins differed significantly. In the P group, proteins involved in oxidative stress-related processes were upregulated, whereas proteins related to salivary secretion were downregulated. The combination of thioredoxin domain-containing protein 17, zymogen granule protein 16B, and FAM3 metabolism regulating signaling molecule D yielded an area under the curve of 0.917 which showed a robust ability to distinguish the P and G groups. In conclusion, poorly controlled hyperglycemia may affect salivary proteins through various pathways, including oxidative stress and glandular secretion. Furthermore, the differentially expressed proteins, especially the three proteins with the best differentiation, might serve as an anchor point for the further study of hyperglycemia and oral diseases.

## 1 Introduction

Diabetes mellitus (DM) is a common metabolic disease manifesting as defects in insulin secretion, insulin action or both, which results in a type of hyperglycemic status. Diabetes mellitus encompasses type 1, type 2, gestational diabetes and other types, and the estimate from the International Diabetes Federation (IDF) is projected to rise to over 640 million people with diabetes by 2040 (Cole and Florez, 2020). Type 2 diabetes mellitus (T2DM) accounts for over 90% of cases of DM worldwide and has become an increasing global public health concern. T2DM can lead to multiple complications, including diabetic neuropathy, diabetic retinopathy, diabetic nephropathy, and cardiovascular complications, which increase disabilities and mortalities in individuals (Zheng et al., 2018).

In addition to the complications of diabetes mentioned above, there are also many oral manifestations associated, including periodontitis, dental caries, oral candidiasis, and salivary dysfunction (Mauri-Obradors et al., 2017). In patients with diabetes, elevated glucose levels contribute to a decrease in the salivary flow rate, a reduction in pH concentration and changes in the oral environment, which easily cause changes in salivary traits (Velasco-Ortega et al., 2016). In addition, diabetic patients suffer from vascular endothelial damage due to specific vascular diseases and are vulnerable to invasion and injury, coupled with an increased systemic inflammatory response in hyperglycemia (Graves et al., 2020). All of these factors promote the occurrence and development of oral complications.

Saliva is a bodily fluid with a complex composition and specific roles. In recent years, salivary proteomics has made great progress, and more than 4,000 protein species have been identified (Siqueira and Dawes, 2011; Huang et al., 2021). Moreover, salivary proteomic research has focused on the screening of novel biomarkers for oral and related diseases, including lactoferrin, interleukin-6, and matrix metalloproteinase-8 (Castagnola et al., 2017; Bermejo-Pareja et al., 2020; Kc et al., 2020; Boroumand et al., 2021). In addition, diabetes affects the composition of saliva, and alterations may impact the incidence or signs of oral lesions (Abd-Elraheem et al., 2017; Naseri et al., 2018). Overall, the impact of diabetes on salivary proteins has attracted the attention of many researchers. In particular, an article on comprehensive proteomic analysis of the human salivary protein in T2DM in 2009 provided the first global view of potential mechanisms reflected in diabetic saliva and their utility in the detection and monitoring of diabetes (Rao et al., 2009). Most studies have focused on the changes in salivary proteins under hyperglycemia and proposed that individual proteins, such as matrix metallopeptidase 2, BPI fold containing family A member 1 and CS pseudogene 1, could be used as breakthroughs for further research (Guo et al., 2017; Arreguin-Cano et al., 2019; Zhang et al., 2020b). To the best of our knowledge, research on the changes in salivary protein groups under different glycemic control states is still relatively limited. The level of glycemic control is closely related to the long-term management of complications of T2DM (Patrick et al., 2021). It is necessary for us to explore the impact of different blood glucose levels on salivary proteomics and have a deeper understanding of changes in the oral environment under poorly controlled hyperglycemia states.

Recently, liquid chromatography coupled with tandem mass spectrometry (LC–MS/MS) has become a powerful tool to detect different biomolecules, for example, proteins and metabolites (Muller et al., 2019). Data-independent acquisition (DIA) is emerging as a powerful tool for proteome quantification which is traditionally different from data-dependent acquisition (DDA) (Venable et al., 2004; Gillet et al., 2012; Doerr, 2015; Ludwig et al., 2018). During the DIA process, it is independent of the composition of precursor ions for their fragmentation to implement the approach, and thus, this method improves the accuracy and precision of quantitation and has gained much popularity (Zhang et al., 2020a). This technique is widely used in various body fluids, including blood, cerebrospinal fluid, urine, and tears but is less applied in saliva (Li et al., 2020; van der Laan et al., 2020; Cheung et al., 2021; Jia et al., 2021).

In this study, we investigated the proteomic profile of whole saliva by LC-MS/MS with the DIA mode in T2DM patients with satisfactory glycemic control in comparison with the poorly-controlled individuals. The aim of the present work was to characterize the salivary proteome at different glucose control levels to identify differentially expressed proteins associated with regulated biological pathways and evaluate potential biomarkers for disease monitoring.

## 2 Materials and Methods

### 2.1 Study Population

The observational research was approved by the Local Ethics Committee at the Peking University School and Hospital of Stomatology (PKUSSIRB-201944042). The clinical register number of this study is ChiCTR1900023582. From April 2019 to July 2019, we recruited 24 subjects, and they all signed written informed consent forms. After matching their periodontal condition and other indicators, they were divided into two groups according to their glycosylated hemoglobin A1c (HbA1c) levels: a well-controlled group (*n* = 8; one male and seven females; HbA1c <6.5%) and a poorly controlled group (n = 16; three males and 13 females; HbA1c ≥6.5%), with no statistical difference in age and sex distribution.

The inclusion criteria were as follows: 1) 50–75 years old; 2) a minimum of 15 teeth (excluding third molars); 3) a clinical diagnosis for at least 1 year with T2DM defined by the latest classification and diagnosis (American Diabetes Association, 2021); and 4) no change in diabetic treatment, including the dosage and formulation, during the last 3 months.

The exclusion criteria were as follows: 1) presence of systemic disease other than T2DM, for example, an acute cardiovascular event in the 12 months prior to the start of the study or stroke, renal failure, or liver dysfunction; 2) presence of the major complications of diabetes; and 3) presence of an active infection such as a periapical abscess.

### 2.2 Clinical Examination

The clinical physicians performed the periodontal examination for all participants using a periodontal probe (UNC15; Hu-Friedy, Chicago, IL, United States). The related parameters were recorded at six sites of each tooth. The measured parameters were as follows: probing pocket depth (PPD), clinical attachment loss (CAL) and bleeding index (BI). All clinical examination procedures were performed by two senior calibrated dentists.

### 2.3 Saliva Sampling and Storage

The participants were requested to refrain from eating, drinking, chewing and brushing for at least 2 h. Each person was asked to rinse their mouth with water for approximately 2 min and then wait 10 min. After that, the participants were taught to collect their whole unstimulated saliva according to a method described by Petros Papagerakis (Papagerakis et al., 2019). The saliva flowed in a sterile 5 ml Eppendorf tube and was transferred to the laboratory on ice. The saliva samples were centrifuged at 10,000 g/min for 10 min, and the supernatants were frozen at −80°C.

### 2.4 Laboratory Assays

#### 2.4.1 Sample Preparation for Proteome Analysis

Samples were suspended in lysis buffer (1% sodium deoxycholate (SDS), 8M urea) which included appropriate 1 protease inhibitor cocktail and phosphatase inhibitor to inhibit protease activity. The mixture was allowed to vortex to mix well and then placed on ice for 30 min during which the sample were vortexed at every 10 min. After centrifugation at 12,000 g at 4°C for 20 min, the concentration of protein supernatant was determined by Bicinchoninic acid (BCA) method by BCA Protein Assay Kit. 50 μg proteins were suspended in 50 μl solution, reduced by adding 1μl 0.5M TCEP incubated at 37°C for 1 h, alkylated by adding 2 μl of 1M iodoacetamide in the dark at room temperature for 40 min. Then the sample was precipitated using 300 µl prechilled acetone at −20°C overnight. The precipitate was washed twice with cold acetone and then resuspended in 50 mM TEAB. Finally, the proteins were digested with trypsin (Promega, Madison, WI) at a substrate/enzyme ratio of 50:1 (w/w) at 37°C for 16 h.

#### 2.4.2 High pH Reverse Phase Separation

The peptide mixture was redissolved and then fractionated by high pH separation using an Ultimate 3000 system (Thermo Fisher Scientific, MA, United States) connected to a reversed-phase column (XBridge C18 column, 4.6 mm × 250 mm, 5 μm, (Waters Corporation, MA, United States)). High pH separation was performed using a linear gradient, starting from 5% B to 45% B in 40 min (B: 20 mm ammonium formate in 80% ACN, pH = 10.0, adjusted with ammonium hydroxide).

#### 2.4.3 Creation of the Spectral Library

To build the spectral library, the peptide solutions were analyzed by nano-HPLC–MS/MS. Specifically, the peptides were redissolved in 30 μl of solvent A (A: 0.1% formic acid in water) and analyzed by online nanospray LC–MS/MS on an Orbitrap Fusion Lumos coupled to an EASY-nLC 1200 system (Thermo Fisher Scientific, MA, United States). A 3 μl peptide sample was loaded onto an analytical column (Acclaim PepMap C18, 75 μm × 25 cm) and separated with a 120-min gradient from 5 to 35% B (B: 0.1% formic acid in ACN).

Raw DDA data were processed and analyzed by Spectronaut X (Biognosys AG, Switzerland) with default settings to generate an initial target list. Spectronaut was set up to search the database of humans along with the contaminant database, assuming trypsin as the digestion enzyme. A q-value (FDR) cutoff at the precursor and protein levels was applied at 1%.

#### 2.4.4 DIA Mode With Nano-HPLC–MS/MS Analysis and Data Acquisition

Each sample was analyzed using the nano-HPLC–MS/MS equipment and LC gradient described above for building the spectral library but using the DIA mode.

Raw DIA data were processed and analyzed by Spectronaut X (Biognosys AG, Switzerland) with default parameters. The retention time prediction type was set to dynamic iRT. Data extraction was determined by Spectronaut X based on extensive mass calibration. Spectronaut Pulsar X will dynamically determine the ideal extraction window depending on iRT calibration and gradient stability. A q-value (FDR) cutoff at the precursor and protein levels was applied at 1%. All selected precursors passing the filters were used for quantification. The average top three filtered peptides that passed the 1% q-value cutoff were used to calculate the major group quantities.

### 2.5 Data Analysis

All statistical analyses were performed using SPSS 24.0 software (SPSS; Chicago, IL, United States). Quantitative variables are described as the mean ± standard deviation (SD), and frequencies or rates were used for qualitative variables. Student’s *t* test was used to compare between-group differences, and a *p*-value <0.5 was defined as statistically significant. Additionally, for qualitative results such as sex, we used the chi-squared test to detect statistically significant differences. After Student’s *t* test, differentially expressed proteins were filtered if their q-value <0.05 and their absolute value of fold change (FC) ≥1.5. Proteins were annotated against the KEGG and GO databases to obtain their functions. Significant pathways and GO functions were examined within differentially expressed proteins with a *p*-value <0.05. In addition, we used the receiver operating characteristic (ROC) curve to screen and evaluate the performance of the model, and the area under the receiver operating characteristic curve (AUC) is a performance index ranging from 0 to 1. AUC is equal to 0.5 when the ROC curve corresponds to random chance and 1.0 for perfect accuracy, while an estimated AUC less than 0.5 represents a worse condition (Zou et al., 2007). A higher AUC value indicates superior discrimination performance.

## 3 Results

### 3.1 Demographic and Clinical Data

In total, 24 individuals diagnosed with T2DM were recruited for the study, and according to their blood glucose level, they were divided into a well-controlled group and a poorly controlled group. The former included eight subjects (G group; mean age 65.25 ± 3.77 years, one male and seven females), and the latter included 16 subjects (P group; mean age 69.69 ± 5.51 years, three males and 13 females). There were no significant differences in age or sex between these two groups (*p*-value = 0.053 and 1.000, respectively). Regarding their clinical status, most indicators were not significantly different except for HBA1c (*p*-value = 0.002) and the fasting plasma glucose (*p*-value <0.001). A total of 24 saliva samples were obtained. More details of these subjects are shown in Table 1.

**TABLE 1:** Characteristics of participants with T2DM.

	Well-controlled group (*n* = 8)	Poorly-controlled group (*n* = 16)	*p*-value
Age (years)	65.25 ± 3.77	69.69 ± 5.51	0.053
Gender(F/M)	7/1	13/3	1.000
Tooth number (count)	26.13 ± 2.30	25.38 ± 2.96	0.750
PPD (mm)	3.27 ± 0.49	3.45 ± 0.44	0.376
AL (mm)	2.39 ± 0.81	3.12 ± 1.04	0.096
BI	2.31 ± 0.88	2.41 ± 0.65	0.756
BOP (+)	0.71 ± 0.27	0.68 ± 0.26	0.815
HbA1c (%)	6.31 ± 0.40	7.79 ± 1.55	0.002*
FPG (mmol/L)	5.93 ± 0.42	8.62 ± 1.52	0.000**
CHOL (mmol/L)	4.86 ± 1.03	4.78 ± 1.14	0.997
TG (mmol/L)	1.64 ± 0.60	1.50 ± 0.82	0.658
HDL (mmol/L)	1.59 ± 0.18	1.37 ± 0.25	0.039
LDL (mmol/L)	2.64 ± 0.96	2.69 ± 0.85	0.835
CRP (mmol/L)	1.39 ± 1.45	2.17 ± 2.10	0.357

Data were described as mean ± SD. PPD, probing pocket depth, Al, attachment level; BI, bleeding index; HbA1c, glycated hemoglobin; FPG, fasting plasma glucose; CHOL, cholesterol; TG, triglyceride; HDL, high-density lipoprotein; LDL, low-density lipoprotein.

*Statistically significant difference (student’s *t* test, *p*-value < 0.05).

**Statistically significant difference (student’s *t* test, *p*-value < 0.001).

### 3.2 Mass Spectrometry Data

**FIGURE 1:**
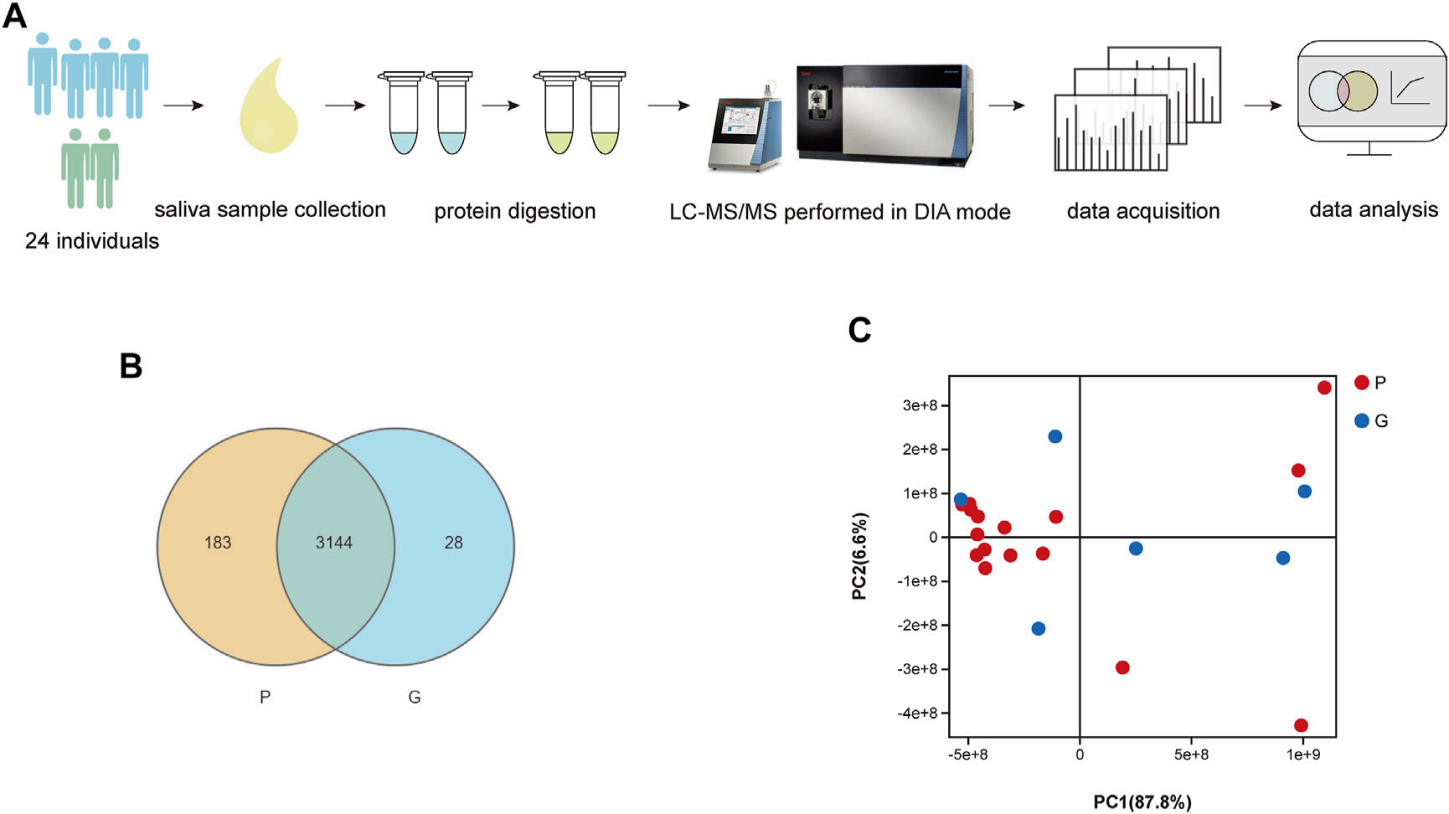
**(A)** Flow diagram of the study design. **(B)** Comparison of number of identified proteins in each sample. Venn-diagram illustrates the sum and percentage of proteins identified in saliva of each group as well as the overlap between the samples. There were 3,144 different proteins identified in samples of two groups. **(C)** PCA analysis of sample relationship. Blue spot indicates the samples of G group, while red spot indicates the P group. PCA plot of samples with G and P groups showed a close distance within each group and a partial separation between groups. The composition of the samples within the group was similar, while the samples were distributed separately between groups, indicating a good agreement and a similarity between those samples in the reduced space.

As shown in Figure 1A, we used LC–MS/MS scanned by the DIA method to quantify and identify proteins in each sample. As a result, the spectral libraries of saliva contained 20,738 precursors, 16,185 peptides, 2,101 protein groups and 5,721 proteins. Following the generation of the library, identification and quantification were implemented. We identified 16,806 precursors, 12,985 peptides, 1,362 protein groups and 3,355 proteins. The sum and percentage of proteins identified in the saliva of each group as well as the overlap between the samples were found in a Venn diagram (Figure 1B). In the P group, 3,327 proteins were quantified, while 3,172 proteins were quantified in the G group. There were 3,144 proteins identified in the two groups. In the PCA plot of sample relationships (Figure 1C), there was a relatively close distance within each group and a partial separation between groups. The composition of the samples within the group was similar, while the samples were distributed separately between groups, indicating a good agreement and a similarity between those samples in the reduced space.

### 3.3 Differentially Expressed Proteins in Saliva

**FIGURE 2:**
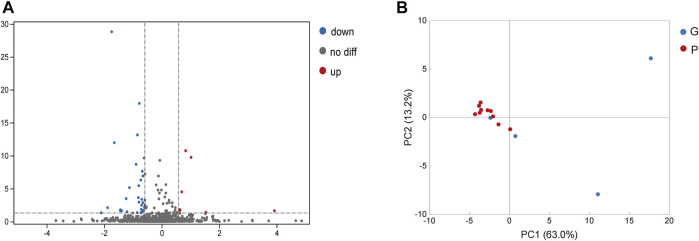
Analysis of differentially expressed proteins in saliva. **(A)** Blue spot indicates the downregulated differently expressed protein, while red spot indicates the upregulated. Black spot represents the proteins with no different expression. Volcano-plot with a 5% FDR and limit of ± 1.5 in fold change in difference of mean quantification. **(B)** Blue spot indicates the differently expressed protein in G group, while red spot indicates the P group. PCA plot of individuals with G and P groups based on the differentially expressed proteins showed a partial separation of the groups.

In saliva samples from the P group, 40 proteins were expressed significantly differently compared to the G group under the screening conditions with a q-value <5% and a limit of fold change (FC) ± 1.5 (Figure 2A). Among these proteins, nine proteins were significantly upregulated in the P group compared to the G group. They included hemoglobin subunit beta, hemoglobin subunit alpha 2, hemoglobin subunit alpha 1, cystatin B and so on (Figure 2A; Table 2). In total, 31 proteins were significantly downregulated in the P group. These proteins included mucin 5B, lactoperoxidase, prolactin-induced protein, etc. (Table 3). The PCA plot of individuals in the G and P groups based on the differentially expressed proteins showed a partial separation of the groups (Figure 2B).

**TABLE 2:** Differentially up-regulated proteins in saliva.

Symbol	Protein name	*p*-value	q-value	AUC
DPYD	Dihydropyrimidine dehydrogenase	0.0013	0.02292	0.955^a^
CA1	Carbonic anhydrase 1	0.0025	0.04052	0.821^a^
TXNDC17	Thioredoxin domain containing 17	0.0007	0.01463	0.802^b^
CDA	Cytidine deaminase	0.0010	0.02045	0.771
S100A11	S100 calcium binding protein A11	0.0009	0.01820	0.677
CSTB	Cystatin B	0.0000	0.00003	0.635
HBA2	Hemoglobin subunit alpha 2	0.0000	0.00000	0.615
HBA1	Hemoglobin subunit alpha 1	0.0000	0.00000	0.615
HBB	Hemoglobin subunit beta	0.0000	0.00000	0.604

a The protein detected in samples with missing values were removed.

b AUC value >0.8.

**TABLE 3:** Differentially down-regulated proteins in saliva.

Symbol	Protein name	*p*-value	q-value	AUC
FAM3D	FAM3 metabolism regulating signaling molecule D	0.0000	0.00034	0.823^a^
ZG16B	Zymogen granule protein 16B	0.0000	0.00000	0.813^a^
FCGBP	Fc fragment of IgG binding protein	0.0000	0.00000	0.792
KRT1	Keratin 1	0.0008	0.01655	0.792
CPE	Carboxypeptidase E	0.0000	0.00000	0.781
PIP	Prolactin induced protein	0.0000	0.00000	0.771
TCN1	Transcobalamin 1	0.0000	0.00043	0.771
CLTC	Clathrin heavy chain	0.0012	0.02292	0.771^b^
TIMP1	TIMP metallopeptidase inhibitor 1	0.0000	0.00136	0.75
PSAP	Prosaposin	0.0017	0.02967	0.75
LPO	Lactoperoxidase	0.0000	0.00000	0.74
WFDC2	WAP four-disulfide core domain 2	0.0003	0.00712	0.729
CA6	Carbonic anhydrase 6	0.0003	0.00795	0.729
CLU	Clusterin	0.0029	0.04507	0.729
DSG3	Desmoglein 3	0.0001	0.00333	0.719
LGALS3BP	Galectin 3 binding protein	0.0000	0.00000	0.708
BPIFB2	BPI fold containing family B member 2	0.0000	0.00000	0.708
NUCB1	Nucleobindin 1	0.0025	0.04052	0.708
KLK11	Kallikrein related peptidase 11	0.0010	0.02045	0.698
CRNN	Cornulin	0.0029	0.04507	0.698
PRR27	Proline rich 27	0.0000	0.00112	0.688
NUCB2	Nucleobindin 2	0.0000	0.00000	0.677
SMR3B	Submaxillary gland androgen regulated protein 3B	0.0000	0.00023	0.677
NUCB2	Nucleobindin 2	0.0000	0.00000	0.677
RNASET2	Ribonuclease T2	0.0033	0.04818	0.677
MUC5B	Mucin 5B, oligomeric mucus/gel-forming	0.0000	0.00000	0.667
CST3	Cystatin C	0.0000	0.00000	0.656
MYH9	Myosin heavy chain 9	0.0006	0.01391	0.646
THBS1	Thrombospondin 1	0.0013	0.02292	0.646
AC018630.2	PRH1-PRR4 readthrough	0.0030	0.04691	0.635
PRH1	Proline rich protein HaeIII subfamily 1	0.0030	0.04691	0.635

a AUC value >0.8.

b The protein detected in samples with missing values were removed.

### 3.4 Functional Annotation of Differentially Expressed Proteins

To investigate the function of differentially expressed proteins, GO and KEGG pathway analyses of upregulated and downregulated proteins were performed separately.

**FIGURE 3:**
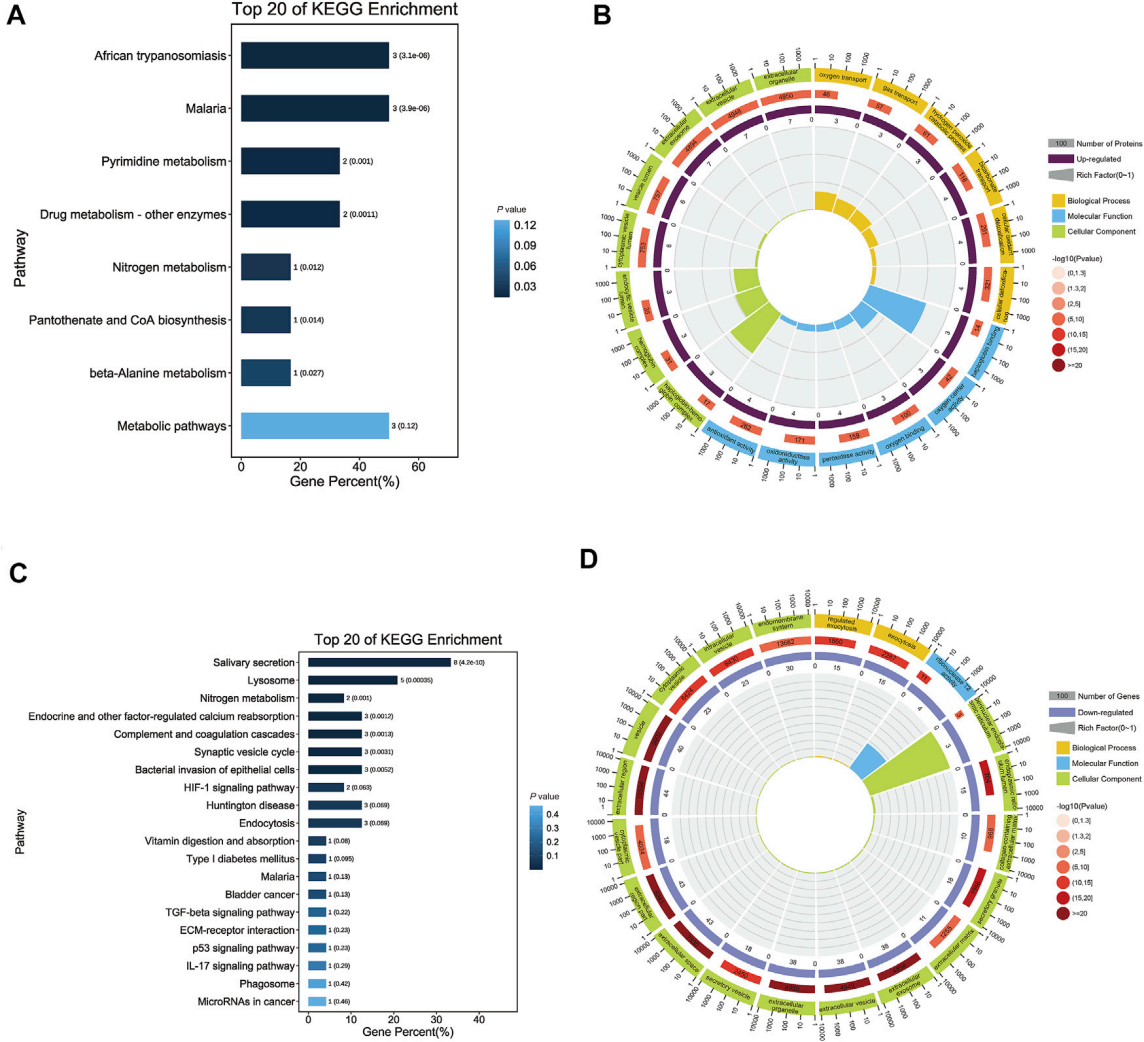
Functional annotation of differentially expressed salivary proteins. **(A)** The bar chart shows the top 20 significantly enriched KEGG pathways that up-regulated proteins were involved. Each bar represents a pathway, the color of the bar represents the enrichment significance of the pathway, and the darker the color is, the smaller the *p*-value is; and the value on the bar is the number of pathways and *p*-value. African trypanosomiasis, Malaria and Metabolic pathways had the same numbers, but the first two had smaller *p*-value. **(B)** The enrichment circle diagram shows the top 20 significantly enriched GO terms that up-regulated proteins were involved. The first circle means the name of pathways and different colors represent different A classes. The second circle shows the number and *p*-value of this pathway in background genes. The more genes, the longer the bar and the smaller the *p*-value, the redder the color. The third circle showed the proportion of up-regulated genes. And the fourth circle showed the rich factor value of each pathway. In GO terms, haptoglobin binding had the highest rich factor. **(C)** The bar chart shows the top 20 significantly enriched KEGG pathways that down-regulated proteins were involved. Each bar represents a pathway, the color of the bar represents the enrichment significance of the pathway, and the darker the color is, the smaller the *p*-value is; and the value on the bar is the number of pathways and *p*-value. Salivary secretion had the most numbers. **(D)** The enrichment circle diagram shows the top 20 significantly enriched GO terms that down-regulated proteins were involved. The first circle means the name of pathways and different colors represent different A classes. The second circle shows the number and *p*-value of this pathway in background genes. The more genes, the longer the bar and the smaller the *p*-value, the redder the color. The third circle showed the proportion of up-regulated genes. And the fourth circle showed the rich factor value of each pathway. In GO terms, perinuclear endoplasmic reticulum had the highest rich factor.

For upregulated proteins, 288 significant enrichments were identified using GO analysis (*p*-value <0.05). GO is a universal resource for analysis and interpretation of high-throughput biological dataset (Kramarz and Lovering, 2019) and we performed GO analysis to obtain the functional characteristics of the differentially expressed proteins and to indicate the GO terms enriched in these proteins. There were three categories: molecular function (MF), biological process (BP) and cellular component (CC). In MF, the proteins were mainly associated with oxidative stress processes, including peroxidase activity, oxidoreductase activity, antioxidant activity and haptoglobin binding. The most significant terms for BP and CC were bicarbonate transport and cytoplasmic vesicle lumen, respectively (Figure 3B). KEGG analysis performed for differentially upregulated proteins showed that the upregulated proteins were significantly enriched in African trypanosomiasis and malaria (Figure 3A).

For downregulated proteins, the functional annotation showed that there were 91 significant GO terms, including ribonuclease T2 activity, low-density lipoprotein particle receptor binding, and endopeptidase inhibitor activity in MF. For BP and CC, the most significant terms were regulated exocytosis and extracellular space (Figure 3D). KEGG pathway analysis revealed that the proteins were significantly enriched in the salivary secretion pathway (Figure 3C).

### 3.5 Analysis of the Discriminating Ability of Candidate Proteins

**FIGURE 4:**
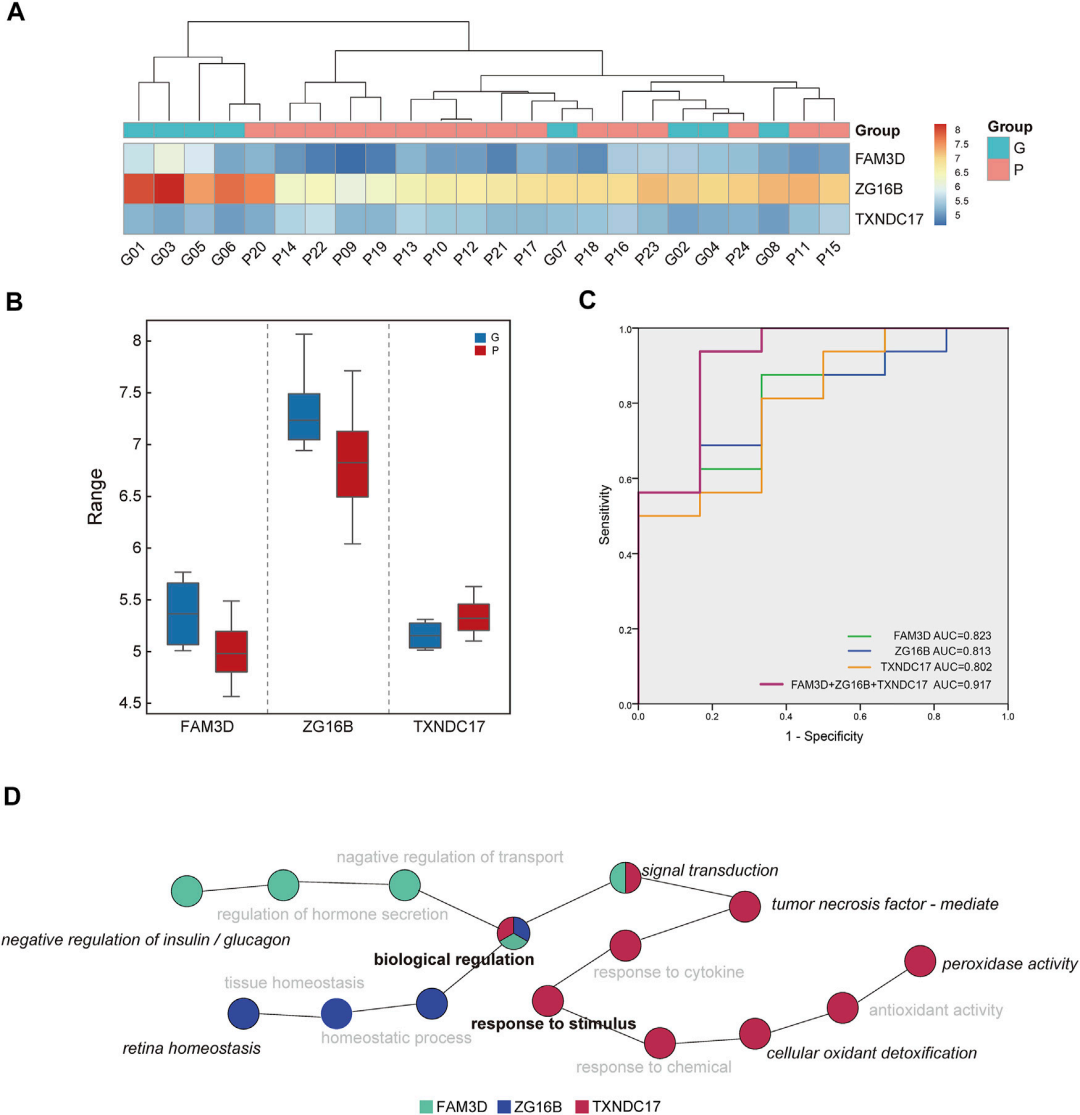
Analysis of discriminating ability of candidate proteins. **(A)** The intensity-based heatmap indicates a partially clear separation of the two groups was observed. There are four samples of G group close to P group after clustering. **(B)** The box diagram shows the comparison of the expression levels of ZG16B, TXNDC17, FAM3D between two groups. **(C)** Receiver operating characteristic (ROC) analysis of candidate proteins in saliva. The combination of three proteins revealed the best performance with an area under the curve of 917. **(D)** The diagram indicates the enriched biological process of three proteins in saliva. Biological regulation and response to stimulus were in the central position, and other specific annotated functions include negative regulation of insulin and glucagon, retina homeostasis, peroxidase activity, etc.

Among the differentially expressed proteins, three proteins with missing values in the samples were removed (Tables 2, 3). Among the remaining proteins, three differentially expressed proteins with high AUC values were selected for further analysis: thioredoxin domain-containing protein 17 (TXNDC17), zymogen granule protein 16B (ZG16B), and FAM3 metabolism regulating signaling molecule D (FAM3D). The expression levels of the three proteins in each sample were used in hierarchical cluster analysis (Figure 4A), which intuitively reflected expression differences between the P and G groups and showed that there was partly clear separation between the two groups. Samples G02, G04, G07, and G08 were far from the other samples in the G group. Generally, there are differences in the abundance of these three proteins between the two glycemic control states, and the sample clusters are relatively distinct.

Compared with the G group, the expression of TXNDC17 increased in saliva, and the other two decreased in the P group. There were significant differences in the expression of TXNDC17, ZG16B and FAM3D (*p*-value <0.05) (Figure 4B).

ROC curve analysis of saliva was performed to verify the differentiation ability of the three proteins. The ability of these proteins to distinguish different HbA1c levels is shown in Figure 4C. The AUCs of each protein ranged from 0.802 to 0.823, whereas the AUC for their combined value was 0.917. ROC curve analysis indicated that the combination of candidate biomarkers in saliva to distinguish the G and P groups had the largest AUC area.

Figure 4D indicates the enriched biological processes of FAM3D, ZG16B, and TXNDC17. They were involved in a variety of functions. GO terms annotated by FAM3D included negative regulation of insulin and glucagon and signal transduction, whereas ZG16B was annotated in retinal homeostasis. TXNDC17 was enriched in biological processes related to the response to stimuli, such as peroxidase activity, cellular oxidant detoxification, and tumor necrosis factor-mediated processes. In addition, all of them were involved in biological regulation and response to stimulus, and thus, the two major functional processes were in the central position.

## 4 Discussion

In this study, 24 patients with T2DM were recruited. After matching their periodontal and demographic status, the subjects were allocated to two groups according to their different control levels of blood glucose. The proteins in the saliva of individuals were detected by LC/LC–MS with DIA scanning. Two groups of differentially expressed proteins were obtained. Through ROC analysis of these proteins, TXNDC17, ZG16B, and FAM3D with high AUC values were finally selected.

In recent years, LC–MS/MS approaches that rely on the DIA method have gained more interest in proteomics because of their advantages in quantification reproducibility, specificity and accuracy, especially the quantification of low protein amounts (Barkovits et al., 2020). However, there are limited studies about DIA applied in oral science. Cecchettini A and Finamore F explored the potential of salivary proteomics to identify primary Sjögren’s syndrome by applying LC-SWATH-MS technology (Cecchettini et al., 2019; Finamore et al., 2021). In our study, we used LC–MS/MS relying on the DIA method to test the subjects’ saliva to ensure the accuracy and coverage of protein quantification and identification. To our knowledge, this study is one of the limited studies collecting saliva to evaluate changes in the proteome of patients with different blood glucose levels by this technology. Undoubtedly, this is a challenge and supplement to apply DIA mass spectrometry technology in the dental field.

As a type of body fluid secreted by the salivary gland, saliva has vast potential for the diagnosis and prognosis of diseases for many reasons, such as its easy, safe, economical and noninvasive collection method (Castagnola et al., 2017). In our report, we found that 40 salivary proteins were expressed at significantly different levels in the P group compared to the G group. Our findings are consistent with those reported in several previous studies (Aitken et al., 2015; Abd-Elraheem et al., 2017; Shirzaiy and Dalirsani, 2019), which indicate that the composition of saliva changes under different blood glucose levels. However, a study involving 84 participants came up with the opposite conclusion (Dodds and Dodds, 1997), which showed that poorly controlled noninsulin-dependent diabetes mellitus had no influence on saliva output. It was worth noting that there were differences between that study and ours in both collection method of saliva (stimulated parotid saliva versus unstimulated whole saliva) and the sample size. Through functional analysis, the upregulated differential proteins were mainly enriched in oxidative stress-associated processes such as peroxidase activity, antioxidant activity, oxidoreductase activity, and oxygen carrier activity, which indicated that elevated and unbalanced oxidative stress states were found in T2DM patients with poor glucose control. A review of salivary gland protein alterations in the diabetic milieu made the same point (Fouani et al., 2021). However, the downregulated differential proteins in our study were enriched in salivary secretion pathways and regulated exocytosis processes, which also confirms a widely held view that salivary glands may be damaged by oxidative stress injury, inflammatory immune response, infection or other reasons secondary to hyperglycemia; thus, secretory function could be impaired (Fouani et al., 2021).

The AUC is used to evaluate the performance of the model (Tang et al., 2019). According to a previous report, a combination of biomarkers improved the predictive value compared with a single biomarker (Wu et al., 2018). When combinations of salivary TXNDC17/ZG16B/FAM3D levels were analyzed, the AUC value was higher than any single one, which means that the combined application of the three in saliva has the most robust ability to distinguish the P and G groups.

TXNDC17 is a novel and highly conserved disulfide reductase that can suppress NF-κB signaling that seems to be related to its inhibition of osteoclast differentiation and bone resorption (Dóka et al., 2020). It is involved in many biological processes, including oxidative stress and redox reactions, specifically peroxidase activity and the reduction of H_2_O_2_ (Jeong et al., 2004). The redox balance within cells and tissues is maintained by antioxidant systems, which are vital to physiological homeostasis. When antioxidant systems are weakened or ROS production is excessive, a state called oxidative stress occurs that will cause direct damage to cells and tissues and active proinflammatory signal transduction. The pathological conditions of diabetes are associated with the generation of oxidative stress (Allen et al., 2009; Yaribeygi et al., 2020). Our results show that in the poorly controlled group, the expression level of TXNDC17 was significantly higher in saliva and that the ability of tissue to reduce ROS production was strengthened. It seems that these people encounter the breakdown of oxidative stress balance and are vulnerable to entering a stressful environment, which makes them more prone to inflammatory reactions and corresponding oral complications (Buczko et al., 2015). Therefore, we can estimate that oxidative stress-related pathways could play a role in the regulation of salivary proteins by poorly controlled hyperglycemia, which is coincident with Masoomeh Shirzaiy, whose report proposed that hyperglycemia and some disturbance in the antioxidant system could cause damage to cell (Shirzaiy and Dalirsani, 2019).

To date, ZG16B (zymogen granule protein 16B) is known as a secretory lectin protein that is proposed to play a regulatory role in lacrimal gland acinar cells to stimulate “flushing out” of the granule content during exocytosis (Kanagawa et al., 2011). To the best of our knowledge, we observed for the first time that the expression of ZG16B was reduced in the saliva of patients with poor control of T2DM. ZG16B has been researched as a biomarker for the diagnosis and progression of many tumors, including pancreatic cancer and colon cancer (Lu et al., 2021), but its detailed biological function is also unknown. It is tempting to suggest that the secretory function of salivary glands under hyperglycemia will be affected, and thus, ZG16B, as a secreted protein, will inevitably be affected and its expression reduced. ZG16B showed little to no protein or transcript expression in other tissues or organs, including the mammary gland and pituitary gland (Saitou et al., 2020), which indicated the tissue expression specificity of ZG16B, and the change in its expression might reflect the change in the secretory function of salivary glands. Studies have also shown that the expression of ZG16B in reflex tears has been detected, and it is speculated that ZG16B may be a critical point in retinal homeostasis and ocular surface protection (Perumal et al., 2015; Perumal et al., 2016), which indicates that the decrease in ZG16B in saliva may indicate the condition of the retina and may even be related to the appearance of ocular lesions. However, the exact relationship of the differential expression of ZG16B in saliva and the occurrence of ocular complications remains to be further studied.

Sequence similarity three member D (FAM3D) is a member of a novel cytokine-like family, the FAM gene family, which is mainly derived from the gut (Zhu et al., 2002). The expression level of FAM3D is affected by nutritional status, with postprandial elevation and a reduction after fasting, and the secretion timing of FAM3D is contrary to that of glucagon (Cao et al., 2017). Some studies found that FAMSD can induce Mac-1-mediated neutrophil recruitment and strongly chemoattract human peripheral blood neutrophils and monocytes (Peng et al., 2016; He et al., 2018), which indicates that FAM3D may be regarded as a proinflammatory factor. Ting Cao et al. found that FAM3D inhibits glucagon secretion via MKP1-dependent suppression of ERK1/2 signaling (Cao et al., 2017). In our research, FAM3D decreased in the poorly controlled group, and we speculate that this change reflects the breakdown of the balance between the proinflammatory and anti-inflammatory states, glucagon and insulin secretion in the body under hyperglycemic conditions.

In recent years, the changes in expression levels and expression patterns of salivary proteins under different disease states and physiological conditions have made them an essential breakthrough in studying the etiology, diagnosis and prognosis of these diseases (Zian et al., 2018; Finamore et al., 2020; Tvarijonaviciute et al., 2020). Changes in salivary protein in states of different blood glucose levels have become a hot spot in the study of the relationship between the oral cavity and diabetes (Bencharit et al., 2013; Lima-Aragão et al., 2016). Alterations in the salivary proteins of T2DM patients have been associated with changes in their metabolism afflicted by chronic hyperglycemia. In recent years, a large number of studies have found many differentially expressed proteins in the state of hyperglycemia, including bone morphogenetic protein 7, lactoferrin, albumin, cathepsin D, etc. (Pappa et al., 2020; Fouani et al., 2021). In our research, we found 40 proteins differentially expressed between the two groups and finally screened out three proteins in saliva that distinguish the two groups well. These three proteins are involved in various functions. We speculate that in the hyperglycemic state, the body’s oxidative stress state, inflammatory state and hormone secretion balance are all disrupted, which causes a variety of macrovascular and microvascular complications, including retinopathy. This hypothesis can also reflect that salivary protein components can show vast aspects of the body affected by hyperglycemia. It will be of inestimable value and potential for further research on the impact of hyperglycemia on the body and oral cavity in the future.

However, there are still some limitations to consider in our study. The first limitation is the absence of subjects with normal glucose conditions. However, the conclusion still has a certain reference value as we have controlled the confounding factors. Second, due to the strict inclusion and exclusion criteria, the sample size is small while there are fewer males in the groups. Although there are no statistical differences in age and sex distribution between the two groups, the unbalanced male and female proportion may also have some impact on the findings. Third, as a cross-sectional study, the present study could not provide strong evidence for a cause-effect relationship. With these limitations, we have to make conclusions and extrapolate the findings with caution. In the future, we need more rigorously designed clinical trials with a larger sample size and healthy controls included for verification and complementation in further.

## 5 Conclusion

In conclusion, the results of this study showed that 40 proteins were differentially expressed in saliva between middle-aged and elderly people with good and poor glycemic control, indicating that, in a hyperglycemic state, oxidative stress state, gland secretion function, hormone regulation, inflammatory response and other processes are all affected. Moreover, the three proteins TXNDC17, ZG16B, and FAM3D showed the best ability to distinguish between the two different states of high and low blood glucose, and their combination could be used to further investigate the in-depth relationship between hyperglycemia and oral disease.

## Data Availability

The data presented in the study are deposited in the ProteomeXchangeConsortium (http://proteomecentral.proteomexchange.org) via the iProX partner repository (Ma et al., 2019) repository, accession number PXD029066.

## References

[B1] Abd-Elraheem S. E. El Saeed A. m. Mansour H. H. (2017). Salivary Changes in Type 2 Diabetic Patients. Diabetes Metab. Syndr. Clin. Res. Rev. 11 (Suppl. 2), S637–s641. 10.1016/j.dsx.2017.04.018 28511885

[B2] Aitken J. P. Ortiz C. Morales-Bozo I. Rojas-Alcayaga G. Baeza M. Beltran C. (2015). α-2-Macroglobulin in Saliva is Associated with Glycemic Control in Patients with Type 2 Diabetes Mellitus. Dis. Markers 2015, 1–5. 10.1155/2015/128653 PMC4363888 25821337

[B3] Allen E. Matthews J. O’Connor R. O'Halloran D. Chapple I. (2009). Periodontitis and Type 2 Diabetes: Is Oxidative Stress the Mechanistic Link? Scott Med. J. 54 (2), 41–47. 10.1258/rsmsmj.54.2.41 19530503

[B4] American Diabetes Association (2021). 2. Classification and Diagnosis of Diabetes: Standards of Medical Care in Diabetes-2021. Dia Care 44 (Suppl. 1), S15–S33. 10.2337/dc21-S002 33298413

[B5] Arreguin-Cano J. A. Ayerdi-Nájera B. Tacuba-Saavedra A. Navarro-Tito N. Dávalos-Martínez A. Emigdio-Vargas A. (2019). MMP-2 Salivary Activity in Type 2 Diabetes Mellitus Patients. Diabetol. Metab. Syndr. 11, 113. 10.1186/s13098-019-0510-2 31892956 PMC6937721

[B6] Barkovits K. Pacharra S. Pfeiffer K. Steinbach S. Eisenacher M. Marcus K. (2020). Reproducibility, Specificity and Accuracy of Relative Quantification Using Spectral Library-Based Data-independent Acquisition. Mol. Cell Proteom. 19 (1), 181–197. 10.1074/mcp.RA119.001714 PMC6944235 31699904

[B7] Bencharit S. Baxter S. S. Carlson J. Byrd W. C. Mayo M. V. Border M. B. (2013). Salivary Proteins Associated with Hyperglycemia in Diabetes: A Proteomic Analysis. Mol. Biosyst. 9 (11), 2785–2797. 10.1039/c3mb70196d 24056972 PMC3888809

[B8] Bermejo‐Pareja F. Del Ser T. Valentí M. de la Fuente M. Bartolome F. Carro E. (2020). Salivary Lactoferrin as Biomarker for Alzheimer’s Disease: Brain‐immunity Interactions. Alzheimer's Demen. 16 (8), 1196–1204. 10.1002/alz.12107 PMC7984071 32543760

[B9] Boroumand M. Olianas A. Cabras T. Manconi B. Fanni D. Faa G. (2021). Saliva, a Bodily Fluid with Recognized and Potential Diagnostic Applications. J. Sep. Sci. 44, 3677–3690. 10.1002/jssc.202100384 34350708 PMC9290823

[B10] Buczko P. Zalewska A. Szarmach I. (2015). Saliva and Oxidative Stress in Oral Cavity and in Some Systemic Disorders. J. Physiol. Pharmacol. 66 (1), 3–9. 25716960

[B11] Cao T. Yang D. Zhang X. Wang Y. Qiao Z. Gao L. (2017). FAM3D Inhibits Glucagon Secretion via MKP1-dependent Suppression of ERK1/2 Signaling. Cell Biol. Toxicol. 33 (5), 457–466. 10.1007/s10565-017-9387-8 28247283

[B12] Castagnola M. Scarano E. Passali G. C. Messana I. Cabras T. Iavarone F. (2017). Salivary Biomarkers and Proteomics: Future Diagnostic and Clinical Utilities. Acta Otorhinolaryngol. Ital. 37 (2), 94–101. 10.14639/0392-100x-1598 28516971 PMC5463528

[B13] Cecchettini A. Finamore F. Ucciferri N. Donati V. Mattii L. Polizzi E. (2019). Phenotyping Multiple Subsets in Sjögren’s Syndrome: a Salivary Proteomic SWATH-MS Approach towards Precision Medicine. Clin. Proteom. 16, 26. 10.1186/s12014-019-9245-1 PMC6587286 31249499

[B14] Cheung J. K.-W. Bian J. Sze Y.-H. So Y.-K. Chow W.-Y. Woo C. (2021). Human Tear Proteome Dataset in Response to Daily Wear of Water Gradient Contact Lens Using SWATH-MS Approach. Data Brief 36, 107120. 10.1016/j.dib.2021.107120 34095372 PMC8165404

[B15] Cole J. B. Florez J. C. (2020). Genetics of Diabetes Mellitus and Diabetes Complications. Nat. Rev. Nephrol. 16 (7), 377–390. 10.1038/s41581-020-0278-5 32398868 PMC9639302

[B16] Dodds M. W. J. Dodds A. P. (1997). Effects of Glycemic Control on Saliva Flow Rates and Protein Composition in Non-insulin-Dependent Diabetes Mellitus. Oral Surg. Oral Med. Oral Pathol. Oral Radiol. Endodontol. 83 (4), 465–470. 10.1016/s1079-2104(97)90147-5 9127379

[B17] Doerr A. (2015). A Bumpy, Holey Method to Probe Proteins. Nat. Methods 12 (1), 14. 10.1038/nmeth.3246 25699317

[B18] Dóka É. Ida T. Dagnell M. Abiko Y. Luong N. C. Balog N. (2020). Control of Protein Function through Oxidation and Reduction of Persulfidated States. Sci. Adv. 6 (1), eaax8358. 10.1126/sciadv.aax8358 31911946 PMC6938701

[B19] Finamore A. Peluso I. Cauli O. (2020). Salivary Stress/Immunological Markers in Crohn’s Disease and Ulcerative Colitis. Int. J. Mol. Sci. 21 (22), 8562. 10.3390/ijms21228562 PMC7698267 33202858

[B20] Finamore F. Cecchettini A. Ceccherini E. Signore G. Ferro F. Rocchiccioli S. (2021). Characterization of Extracellular Vesicle Cargo in Sjögren’s Syndrome Through a SWATH-MS Proteomics Approach. Int. J. Mol. Sci. 22 (9), 4864. 10.3390/ijms22094864 34064456 PMC8124455

[B21] Fouani M. Basset C. A. Jurjus A. R. Leone L. G. Tomasello G. Leone A. (2021). Salivary Gland Proteins Alterations in the Diabetic Milieu. J. Mol. Histol. 52, 893–904. 10.1007/s10735-021-09999-5 34212290 PMC8487876

[B22] Gillet L. C. Navarro P. Tate S. Röst H. Selevsek N. Reiter L. (2012). Targeted Data Extraction of the MS/MS Spectra Generated by Data-independent Acquisition: a New Concept for Consistent and Accurate Proteome Analysis. Mol. Cell. Proteom. 11 (6), O111.016717. 10.1074/mcp.O111.016717 PMC3433915 22261725

[B23] Graves D. T. Ding Z. Yang Y. (2020). The Impact of Diabetes on Periodontal Diseases. Periodontology 82 (1), 214–224. 10.1111/prd.12318 31850631

[B24] Guo Y. Guo L.-N. Zhu J.-F. Tang C.-Y. Feng Y.-Z. Zhou H.-D. (2017). Associations of Salivary BPIFA1 Protein in Chronic Periodontitis Patients with Type 2 Diabetes Mellitus. Int. J. Endocrinol. 2017, 1–13. 10.1155/2017/1087017 PMC5646319 29109737

[B25] He L. Fu Y. Deng J. Shen Y. Wang Y. Yu F. (2018). Deficiency of FAM3D (Family With Sequence Similarity 3, Member D), A Novel Chemokine, Attenuates Neutrophil Recruitment and Ameliorates Abdominal Aortic Aneurysm Development. Arterioscler. Thromb. Vasc. Biol. 38 (7), 1616–1631. 10.1161/atvbaha.118.311289 29853563 PMC6039426

[B26] Huang L. Shao D. Wang Y. Cui X. Li Y. Chen Q. (2021). Human Body-Fluid Proteome: Quantitative Profiling and Computational Prediction. Brief Bioinform. 22 (1), 315–333. 10.1093/bib/bbz160 32020158 PMC7820883

[B27] Jeong W. Chang T.-S. Boja E. S. Fales H. M. Rhee S. G. (2004). Roles of TRP14, a Thioredoxin-Related Protein in Tumor Necrosis Factor-α Signaling Pathways. J. Biol. Chem. 279 (5), 3151–3159. 10.1074/jbc.M307959200 14607843

[B28] Jia L. Wu J. Wei J. Du L. Wang P. Zhang Y. (2021). Proteomic Analysis of Urine Reveals Biomarkers for the Diagnosis and Phenotyping of Abdominal-Type Henoch-Schonlein Purpura. Transl Pediatr. 10 (3), 510–524. 10.21037/tp-20-317 33850810 PMC8039785

[B29] Kanagawa M. Satoh T. Ikeda A. Nakano Y. Yagi H. Kato K. (2011). Crystal Structures of Human Secretory Proteins ZG16p and ZG16b Reveal a Jacalin-Related β-prism Fold. Biochem. Biophys. Res. Commun. 404 (1), 201–205. 10.1016/j.bbrc.2010.11.093 21110947

[B30] Kc S. Wang X. Z. Gallagher J. E. (2020). Diagnostic Sensitivity and Specificity of Host‐derived Salivary Biomarkers in Periodontal Disease Amongst Adults: Systematic Review. J. Clin. Periodontol. 47 (3), 289–308. 10.1111/jcpe.13218 31701554

[B31] Kramarz B. Lovering R. C. (2019). Gene Ontology: A Resource for Analysis and Interpretation of Alzheimer’s Disease Data. Editor Wisniewski T. (Brisbane: Codon Publications). 31895510

[B32] Li K. W. Gonzalez-Lozano M. A. Koopmans F. Smit A. B. (2020). Recent Developments in Data Independent Acquisition (DIA) Mass Spectrometry: Application of Quantitative Analysis of the Brain Proteome. Front. Mol. Neurosci. 13, 564446. 10.3389/fnmol.2020.564446 33424549 PMC7793698

[B33] Lima-Aragão M. V. V. de Oliveira-Junior J. d. J. Maciel M. C. G. Silva L. A. do Nascimento F. R. F. Guerra R. N. M. (2016). Salivary Profile in Diabetic Patients: Biochemical and Immunological Evaluation. BMC Res. Notes 9, 103. 10.1186/s13104-016-1881-1 26879274 PMC4754859

[B34] Lu H. Shi C. Liu X. Liang C. Yang C. Wan X. (2020). Identification of ZG16B as a Prognostic Biomarker in Breast Cancer. Open Med. 16 (1), 1–13. 10.1515/med-2021-0004 PMC7718615 33336077

[B35] Ludwig C. Gillet L. Rosenberger G. Amon S. Collins B. C. Aebersold R. (2018). Data‐Independent Acquisition‐based SWATH ‐ MS for Quantitative Proteomics: A Tutorial. Mol. Syst. Biol. 14 (8), e8126. 10.15252/msb.20178126 30104418 PMC6088389

[B36] Ma J. Chen T. Wu S. Yang C. Bai M. Shu K. (2019). iProX: an Integrated Proteome Resource. Nucleic Acids Res. 47 (D1), D1211–d1217. 10.1093/nar/gky869 30252093 PMC6323926

[B37] Mauri-Obradors E. Estrugo-Devesa A. Jane-Salas E. Vinas M. Lopez-Lopez J. (2017). Oral Manifestations of Diabetes Mellitus. A Systematic Review. Med. Oral 22 (5), e586–e594. 10.4317/medoral.21655 PMC5694181 28809366

[B38] Müller F. Kolbowski L. Bernhardt O. M. Reiter L. Rappsilber J. (2019). Data-Independent Acquisition Improves Quantitative Cross-Linking Mass Spectrometry. Mol. Cell Proteom. 18 (4), 786–795. 10.1074/mcp.TIR118.001276 PMC6442367 30651306

[B39] Papagerakis P. Zheng L. Kim D. Said R. Ehlert A. A. Chung K. K. M. (2019). Saliva and Gingival Crevicular Fluid (GCF) Collection for Biomarker Screening. Methods Mol. Biol. 1922, 549–562. 10.1007/978-1-4939-9012-2_41 30838599

[B40] Pappa E. Vougas K. Zoidakis J. Vastardis H. (2020). Proteomic Advances in Salivary Diagnostics. Biochim. Biophys. Acta Proteins Proteom. 1868 (11), 140494. 10.1016/j.bbapap.2020.140494 32663525

[B41] Patrick N. B. Yadesa T. M. Muhindo R. Lutoti S. (2021). Poor Glycemic Control and the Contributing Factors Among Type 2 Diabetes Mellitus Patients Attending Outpatient Diabetes Clinic at Mbarara Regional Referral Hospital, Uganda. Diabetes Metab. Syndr. Obes. 14, 3123–3130. 10.2147/dmso.S321310 34262316 PMC8275135

[B42] Peng X. Xu E. Liang W. Pei X. Chen D. Zheng D. (2016). Identification of FAM3D as a Novel Endogenous Chemotaxis Agonist for the FPRs (Formyl Peptide Receptors). J. Cell Sci. 129 (9), 1831–1842. 10.1242/jcs.183053 26966188

[B43] Perumal N. Funke S. Pfeiffer N. Grus F. H. (2016). Proteomics Analysis of Human Tears from Aqueous-Deficient and Evaporative Dry Eye Patients. Sci. Rep. 6, 29629. 10.1038/srep29629 27436115 PMC4951640

[B44] Perumal N. Funke S. Wolters D. Pfeiffer N. Grus F. H. (2015). Characterization of Human Reflex Tear Proteome Reveals High Expression of Lacrimal Proline-Rich Protein 4 (PRR4). Proteomics 15 (19), 3370–3381. 10.1002/pmic.201400239 26173177

[B45] Rao P. V. Reddy A. P. Lu X. Dasari S. Krishnaprasad A. Biggs E. (2009). Proteomic Identification of Salivary Biomarkers of Type-2 Diabetes. J. Proteome Res. 8 (1), 239–245. 10.1021/pr8003776 19118452

[B46] Sadeghi M. Naseri R. Mozaffari H. Ramezani M. (2018). Effect of Diabetes Mellitus Type 2 on Salivary Glucose, Immunoglobulin A, Total Protein, and Amylase Levels in Adults: A Systematic Review and Meta-Analysis of Case-Control Studies. J. Res. Med. Sci. 23, 89. 10.4103/jrms.JRMS_135_18 30505327 PMC6225459

[B47] Saitou M. Gaylord E. A. Xu E. May A. J. Neznanova L. Nathan S. (2020). Functional Specialization of Human Salivary Glands and Origins of Proteins Intrinsic to Human Saliva. Cell Rep. 33 (7), 108402. 10.1016/j.celrep.2020.108402 33207190 PMC7703872

[B48] Shirzaiy M. Dalirsani Z. (2019). The Effect of Glycemic Control on Salivary Lipid Peroxidation in Type II Diabetic Patients. Diabetes Metab. Syndr. Clin. Res. Rev. 13 (3), 1991–1994. 10.1016/j.dsx.2019.04.004 31235125

[B49] Siqueira W. L. Dawes C. (2011). The Salivary Proteome: Challenges and Perspectives. Prot. Clin. Appl. 5 (11–12), 575–579. 10.1002/prca.201100046 21956964

[B50] Tang H. Yuan C. Ma Z. Zhu C. Tong P. Gallagher J. E. (2019). The Potentiality of Salivary Peptide Biomarkers for Screening Patients with Periodontal Diseases by Mass Spectrometry. Clin. Chim. Acta 495, 278–286. 10.1016/j.cca.2019.04.076 31026423

[B51] Tvarijonaviciute A. Zamora C. Ceron J. J. Bravo-Cantero A. F. Pardo-Marin L. Valverde S. (2020). Salivary Biomarkers in Alzheimer’s Disease. Clin. Oral Invest. 24 (10), 3437–3444. 10.1007/s00784-020-03214-7 31989369

[B52] van der Laan T. Boom I. Maliepaard J. Dubbelman A.-C. Harms A. C. Hankemeier T. (2020). Data-Independent Acquisition for the Quantification and Identification of Metabolites in Plasma. Metabolites 10 (12), 514. 10.3390/metabo10120514 PMC7766927 33353236

[B53] Velasco-Ortega E. Delgado-Ruiz R. A. López-López J. (2016). Dentistry and Diabetes: The Influence of Diabetes in Oral Diseases and Dental Treatments. J. Diab. Res. 2016, 1. 10.1155/2016/6073190 PMC5227176 28119931

[B54] Venable J. D. Dong M.-Q. Wohlschlegel J. Dillin A. Yates J. R. (2004). Automated Approach for Quantitative Analysis of Complex Peptide Mixtures from Tandem Mass Spectra. Nat. Methods 1 (1), 39–45. 10.1038/nmeth705 15782151

[B55] Wu Y.-C. Ning L. Tu Y.-K. Huang C.-P. Huang N.-T. Chen Y.-F. (2018). Salivary Biomarker Combination Prediction Model for the Diagnosis of Periodontitis in a Taiwanese Population. J. Formos. Med. Assoc. 117 (9), 841–848. 10.1016/j.jfma.2017.10.004 29129647

[B56] Yaribeygi H. Sathyapalan T. Atkin S. L. Sahebkar A. (2020). Molecular Mechanisms Linking Oxidative Stress and Diabetes Mellitus. Oxid. Med. Cell Longev. 2020, 1–13. 10.1155/2020/8609213 PMC7085395 32215179

[B57] Zhang F. Ge W. Ruan G. Cai X. Guo T. (2020a). Data‐Independent Acquisition Mass Spectrometry‐Based Proteomics and Software Tools: A Glimpse in 2020. Proteomics 20 (17–18), 1900276. 10.1002/pmic.201900276 32275110

[B58] Zhang L. Wang H. Jin H. Kim J. Oh S. (2020b). Common Salivary Protein 1 in Saliva of Diabetes Patients (II). Clin. Lab. 66 (12), 2495–2502. 10.7754/Clin.Lab.2020.200327 33337828

[B59] Zheng Y. Ley S. H. Hu F. B. (2018). Global Aetiology and Epidemiology of Type 2 Diabetes Mellitus and its Complications. Nat. Rev. Endocrinol. 14 (2), 88–98. 10.1038/nrendo.2017.151 29219149

[B60] Zhu Y. Xu G. Patel A. McLaughlin M. M. Silverman C. Knecht K. A. (2002). Cloning, Expression, and Initial Characterization of a Novel Cytokine-like Gene Family. Genomics 80 (2), 144–150. 10.1006/geno.2002.6816 12160727

[B61] Zian Z. Bakkach J. Barakat A. Ghailani Nourouti N. Bennani Mechita M. (2018). Salivary Biomarkers in Systemic Sclerosis Disease. Biomed. Res. Int. 2018, 1–7. 10.1155/2018/3921247 PMC5867662 29721505

[B62] Zou K. H. O’Malley A. J. Mauri L. (2007). Receiver-Operating Characteristic Analysis for Evaluating Diagnostic Tests and Predictive Models. Circulation 115 (5), 654–657. 10.1161/circulationaha.105.594929 17283280

